# Supplementary figures and images for: In vitro–in silico correlation of three-dimensional turbulent flows in an idealized mouth-throat model
Source: PLoS Comput Biol. 2023 Mar 23;19(3):e1010537. doi: 10.1371/journal.pcbi.1010537 (PMC10072468; doi:10.1371/journal.pcbi.1010537)

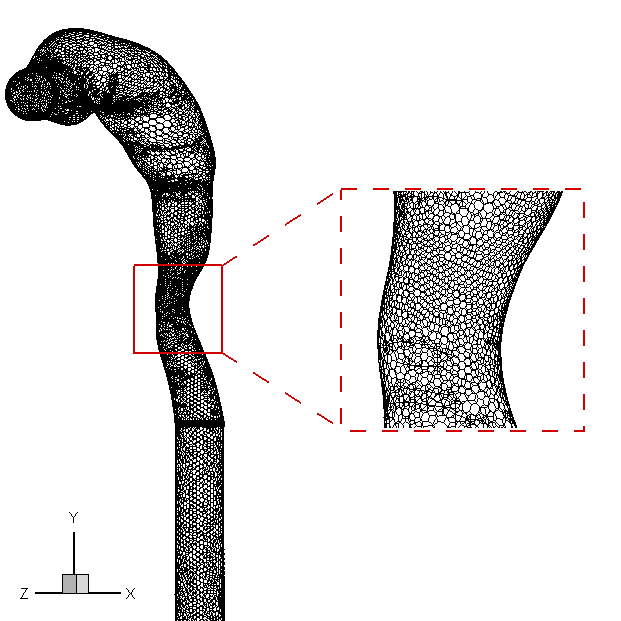

Supplement: S2 Fig — (TIFF) [file pcbi.1010537.s002.tiff]
