# Supplementary material for: In vitro–in silico correlation of three-dimensional turbulent flows in an idealized mouth-throat model
Source: PLoS Comput Biol. 2023 Mar 23;19(3):e1010537. doi: 10.1371/journal.pcbi.1010537 (PMC10072468; doi:10.1371/journal.pcbi.1010537)
Supplement: S1 Text — (PDF) [file pcbi.1010537.s004.pdf]

Supplementary Material: RANS Equations

The kinematic eddy viscosity in SST k-ω model is defined as:

$$\nu_T = \frac{a_1 k}{\max\left(a_1 \omega, S F_2\right)} \tag{1}$$

The transport equation for turbulent kinetic energy is defined as:

$$\frac{\partial k}{\partial t} + U_j \frac{\partial k}{\partial x_j} = P_k - \beta^* k \omega + \frac{\partial}{\partial x_j}\left[\left(\nu + \sigma_k \nu_T\right)\frac{\partial k}{\partial x_j}\right] \tag{2}$$

The transport equations for specific turbulent dissipation rate are given as:

$$\frac{\partial \omega}{\partial t} + U_j \frac{\partial \omega}{\partial x_j} = \alpha S^2 - \beta \omega^2 + \frac{\partial}{\partial x_j}\left[\left(\nu + \sigma_\omega \nu_T\right)\frac{\partial \omega}{\partial x_j}\right] \tag{3}$$

$$+2\left(1 - F_1\right)\sigma_{\omega 2}\frac{1}{\omega}\frac{\partial k}{\partial x_i}\frac{\partial \omega}{\partial x_i} \tag{4}$$

where, the model coefficients and relations can be defined as given below:

$$F_2 = \tanh\left[\left[\max\left(\frac{2\sqrt{k}}{\beta^* \omega y}, \frac{500\nu}{y^2 \omega}\right)\right]^2\right] \tag{5}$$

$$P_k = \min\left(\tau_{ij}\frac{\partial U_i}{\partial x_j}, 10\beta^* k \omega\right) \tag{6}$$

$$F_1 = \tanh\left\{\left\{\min\left[\max\left(\frac{\sqrt{k}}{\beta^* \omega y}, \frac{500\nu}{y^2 \omega}\right), \frac{4\sigma_{\omega 2} k}{CD_{k\omega} y^2}\right]\right\}^4\right\} \tag{7}$$

$$CD_{k\omega} = \max\left(2\rho\sigma_{\omega 2}\frac{1}{\omega}\frac{\partial k}{\partial x_i}\frac{\partial \omega}{\partial x_i}, 10^{-10}\right) \tag{8}$$

$$\varphi = \varphi_1 F_1 + \varphi_2 (1 - F_1) \tag{9}$$

$$\alpha_1 = \frac{5}{9}, \alpha_2 = 0.44 \tag{10}$$

$$\beta_1 = \frac{3}{40}, \beta_2 = 0.0828 \tag{11}$$

$$\beta^* = \frac{9}{100} \tag{12}$$

$$\sigma_{k1} = 0.85, \sigma_{k2} = 1 \tag{13}$$

$$\sigma_{\omega 1} = 0.5, \sigma_{\omega 2} = 0.856 \tag{14}$$

where ν and ν_T are deined as the kinematic viscosity and the turbulent viscosity.
