# Supplementary material for: In vitro–in silico correlation of three-dimensional turbulent flows in an idealized mouth-throat model
Source: PLoS Comput Biol. 2023 Mar 23;19(3):e1010537. doi: 10.1371/journal.pcbi.1010537 (PMC10072468; doi:10.1371/journal.pcbi.1010537)
Supplement: Attachment — Submitted filename: Nof_PLoSCompBiol_Rebuttal.pdf [file pcbi.1010537.s007.pdf]

Ref.: Ms. No. PCOMPBIOL-D-22-01317
In vitro–in silico correlation of three-dimensional turbulent flows in an idealized mouth-throat model

We are pleased to submit our revised manuscript entitled "*In vitro–in silico* correlation of three-dimensional turbulent flows in an idealized mouth-throat model" for publication in *PLoS Computational Biology*. We thank both Reviewers for their feedback and sharing their input in providing detailed comments to help improve and strengthen the scope of our manuscript. We have made efforts to accommodate the Reviewers' suggestions and address their concerns. Corresponding changes in the manuscript have been highlighted for clarity (i.e. new text included is shown in red). We detail below our response to each Reviewer.

Reviewer 1:
The study by Nof et al. provides exciting 3D maps of velocity fields from Tomographic Particle Image Velocimetry (TPIV), RANS simulations, and LES simulations of an idealized mouth-throat geometry of a healthy adult. This is the first study to provide 3D TPIV measurements, which can be used to validate computational models. Notably, the authors provide the 3D data through an online repository, which can be used by the community. This is a well written paper and I have just a few minor comments that should be addressed prior to publication.

Response: We thank the Reviewer for the positive response and acknowledging our efforts in providing further needed benchmark measurements, in conjunction with state-of-the-art numerical simulations, to help serve the scientific community in establishing gold standards. Below, we detail changes to the manuscript according to the points raised by the Reviewer.

1. Thank you for providing the particle relaxation time. Can you provide us with how much a particle is expected to drift during an experiment?

    Response: We thank the Reviewer for raising this comment. We recall that our experiments are conducted in a water/glycerol solution (i.e. 52/48 mass ratio) based on dynamic similarity considerations for PIV measurements. While the particle relaxation time in our experimental setup is much shorter than the characteristic time scale of the flow (i.e. St<<1), as also detailed in Nof *et al.* (2020), we assume that with "drift" the Reviewer implies possible effects of buoyancy of the particles due to density differences between the working fluid (1'150 kg/m^3) and the particle (1'050 kg/m^3), respectively. As such, the (positive) terminal velocity is $u_t = gd_p^2 (\rho_f - \rho_p)/18\mu_f$ ~ 5.7 × 10^{-7} m/s (where the dynamic viscosity of the working fluid is μ_f = 9.66 × 10^{-3} kg/m-s). This buoyant velocity is calculated to be ~0.57 µm/s and therefore overwhelmingly negligible relative to the characteristic velocities of the flow, i.e. *O*(1) m/s. In particular, over the time scale of the PIV measurements (i.e. second), this displacement is negligible.

Following these remarks, we have now added such discussions in the revised methods section (see new paragraph in Experimental Methods section).

2. The no-slip wall boundary condition is hard to observe on the cutouts in Fig. 3. Is this because of near wall averaging of the experimental data?

   Response: The Reviewer is correct to point out there are limited PIV data in the vicinity of the model's walls. This is a well-known and intrinsic limitation of general PIV techniques (and thus, typically adding to the usefulness of CFD simulations) and is generally due to the decreasing concentration (or loss) of tracer particles at the walls, in conjunction with significant flow gradients resulting from the no-slip condition at the wall. Following the Reviewer's comment, we have now added a short discussion of this limitation in the "Global jet characteristics" section (i.e. see end of first paragraph).

3. It would be great to include a brief discussion on how the flow profile differences between RANS and LES might lead to differences in particle deposition. Specifically, is it expected that the unresolved TKE might under-estimate deposition?

   Response: We thank the Reviewer for raising this point and discussing the ramifications of the differences in the simulation results with regards to aerosol transport/deposition in the lungs. For example, the lower TKE levels obtained with RANS are acknowledged to lead to overpredictions of particle deposition when only the (time-averaged) RANS velocity field is used (this is discussed for example in Koullapis *et al.* 2018 and Matilda *et al.* 2004). Following the Reviewer's comments, we have added a brief discussion on this point in the revised 3rd paragraph of the "Glottal jet characteristics'" sub-section in the Results and Discussion, and referenced the relevant literature.

Reviewer #2:

The manuscript describes a comparison of Tomogaphic (3D) PIV measurements with LES and RANS in a mouth throat model. Although the authors report about limitations of already existing studies, their results do not much contribute to new knowledge. It is rather another Benchmark study which shows good agreement between LES and TPIV and larger deviations for the RANS simulations as already reported in their reference [23 - Koullapis et al.]. The advantage of 3D measurements which was emphasized as new in the paper could not be found. The authors showed again detailed comparisons for the mid-sagittal plane and one cross section. The results could have been received also from Stereo PIV (if pure secondary flows had been presented in Fig. 5, 2D planar PIV would have been sufficient to gain the same results). Although additional planes are roughly shown in Fig. 3, their details are not given or discussed. Thus, introduction and conclusion should be changed accordingly.

Response: We thank the Reviewer for the provided feedback to help improve the scope of the manuscript. We note, nevertheless, that while the figures shown here (e.g. Fig. 5) exemplify 2D cut planes, it is important to emphasize that our work includes the 3D velocity vector field measured from TPIV (and shared in the repository; see last response point); this strongly contrasts with e.g. stereo-PIV data that would be nearly impossible to reproduce our data and underlines the uniqueness of our benchmark paper as highlighted at the end of the introduction and conclusions. With this in mind, we provide below detailed answers to each of the Reviewer's points (see also changes to the manuscript's text accordingly).

1. The introduction is too long and should concentrate on the main goal of the paper

   Response: Following the Reviewer's suggestion, we have made significant efforts to reduce the overall length of the introduction (i.e. we reduced by nearly a page with an introduction now standing at ~1.5 pages), while importantly maintaining the original comprehensiveness to give the reader sufficient background on all the relevant numerical and experimental studies available in the literature to date (note that the deleted sentences do not appear in red in the text).

2. Important information in the description of the experimental methods are missing. These are

   - The time delay between successive images used for the PIV measurements (for all 3 flow rates)
     Response: Thank you for the comment. In the present work, image acquisition was conducted using a single frame mode such that the time delay is directly calculated from the frame rate (i.e. delta_t = 1/fps), namely we have used 1'250 fps during acquisition. These details have now been added to the Methods section for consistency.

   - What was the field of view (FOV) in mm and what pixel and vector resolution could have been achieved (see next comment)? The size of the FOV (for each camera) is probably something in the range of 100 x 80 mm^2. With the camera pixels given, one would achieve a resolution of about 80 µm/Px. It is mentioned that tracers in the size of 10µm have been used. That means, they do not even cover a pixel. How were the measurements possible ad more important, what errors/uncertainties can be expected? Peak locking must have been a big issue. Please elaborate on these discrepancies. This becomes furthermore important when differences to the CFD results are discussed.
     Response: We thank the Reviewer for giving the opportunity to clarify this point. We are well aware of peak locking (i.e. where particles appear as single pixels). Notably, this work is in line with our previous efforts involving tomo-

PIV methods (see e.g. Nof et al. J. Roy Soc. 2020 and Nof et al. Bioeng. Transl. Biomed. 2021) where we have painstakingly and rigorously optimized the seeding conditions to guarantee that individual particles in the focal depth of the camera correspond to ~3-5 pixels (i.e. z=0 plane). Overwhelmingly, TPIV and numerical data are well in line (e.g. see 1D velocity comparisons in Fig. 6). Below we show an instantaneous snapshot of a zoom-in showing individual particles (overlapping over several pixels). Following the Reviewer's comment we have amended the methods section to provide additional details pertaining to the TPIV method where we note that the FOV is 18.5 x 45 x 9 mm (X-Y-Z).

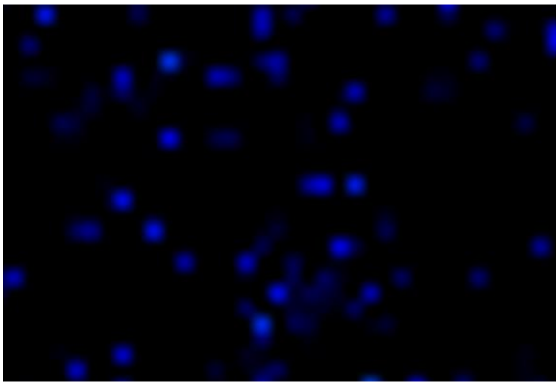

- What was the reason for the use of fluorescent tracers? – as no optical filters have been mentioned.
  Response: We thank the Reviewer for the comment. Indeed, optical filters were fitted to each camera lens to reduce non-fluorescent light reflection. We recall that fluorescent particles are commonly used in PIV to increase contrast and reduce the signal-to-noise ratio (SNR). Such remarks have now been added in the new paragraph of the Methods section.

- Obviously, the side walls of the mouth throat model were not illuminated anymore but the light was cut off by the knife edges. Please explain why this was done as flow information seems to be missing then for Fig. 5.
  Response: We thank the Reviewer for the comment. We recall that knife edges are commonly used in PIV to reduce light reflections from regions without tracer particles, thereby contributing to the rectangularity of the processed vector maps relative to the actual elliptical shape of the experimental model. The near-wall regions are, in any case, heavily skewed in PIV due to strong gradients and loss of particles near the wall, an inherent feature of the PIV method. These remarks have now been added both in the revised Methods section and in the Results section.

3. RANS details: the lines 163 – 181 only repeat texbook knowledge. This passage can be omitted.

Response: We are thankful for the Reviewer's remark suggesting such modification. To maintain strong consistency and the article's general aim to be a self-contained benchmark exemplifying the *in vitro* and *in silico* techniques, we have kept the main details of this paragraph in the main text for readers who are less familiar with the basic details of RANS and specifically, the k-omega-SST model (note that the equations are kept in the article's Appendix section).

4. Fig 1: The camera lenses are not oriented perpendicular to the phantom model wall. Please elaborate on possible errors due to optical distortions. E.g. Buchmann et al. (Exp Fluids (2011) 50:1131–1151) have used prisms to exclude any optical distortions. Why were these not necessary here?

Response: We thank the Reviewer for the remark. In general, the Reviewer is right. Specifically, in our experimental setup the angles are small such that wall reflections were minimal. Furthermore, a critical 3D volume calibration step was first done using a 3D calibration plate (and following Wieneke et al. 2008) to account for refractive index mismatching leading to optical distortions, which Buchmann et al. similarly solved using physical prisms. The details of our volume calibration are provided in Ref. [35], which we refer to in the second paragraph of the Methods section.

5. Fig. 3 and 4: The mid-sagittal plane was significantly wider for the TPIV than for CFD. Please elaborate

Response: We thank the opportunity to clarify this latter point and note that the observed deviation is nevertheless on the order of errors introduced by the volume calibration step and is likely also influenced by the physical compliance of the silicone phantom model. We underline that these small deviations do not under any circumstance affect the main results and conclusions of the manuscript. Following such remarks, we have now added clarification and discussion in the revised Results section when elaborating on Fig. 4 (and correspondingly Fig. 3), where the final physical scales deviate on the order of <5%.

6. Secondary Flows: In this section Fig. 5 should show secondary flow. However, color coded is again the velocity magnitude. It would be more interesting to visualize only the in plane velocity components to show only the secondary flow. The described dean vortices can be identified only with difficulty.

Response: We thank the Reviewer for raising this point. In view of better visualizing the existence of secondary flows, we have now removed as suggested the background color-coding of the velocity magnitude and instead added the reconstructed streamlines for the experimental/numerical data in an effort to underline the presence of Dean-like vortices. Figure 5 has now been updated accordingly (including the caption).

7. There must be something wrong with the scaling in Fig. 5 for TPIV. It is mentioned though that only a rectangular cross-sectional view could be shown here, but the dimensions do not agree at all with the CFD model. The horizontal extension is much wider than the actual model geometry, the vertical structures seem to be squeezed. the vertical extension of the low speed region is much smaller for PIV than for the CFD results. At least the characteristic round geometry cannot be recognized for PIV. This needs correction.

Response: We thank the Reviewer for the comment. We recall (see also above responses) that there are inherent challenges in PIV that introduce physical size errors relative to the CFD geometry, including compliance with the phantom model, the use of knife edges, the volume self-calibration algorithm, and dependence on heterogeneous particle distribution (a feature of the flow) for the reconstruction of the volume. These comments have now been added to clarify that the final physical scales deviate on the order of <5% relative to the CFD geometry, which does not affect the paper's main thrust. In parallel, we have also prepared a new Fig. 5 (following the comment above) that also includes the revised scaling of the plots.

8. In the suppl. Mat. S3 Files, the authors mention a repository for the measured 3D vector field source files. These data could not be found. Please add or correct this.

Response: We thank the Reviewer for the correction. The correct link is now provided here for the reviewers (whereas the final link as we had provided in the manuscript will be made available upon publication):
https://figshare.com/s/5580fa58e6c7698824ec
